# Dog facial landmarks detection and its applications for facial analysis

**DOI:** 10.1038/s41598-025-07040-3

**Published:** 2025-07-01

**Authors:** George Martvel, Anna Zamansky, Giulia Pedretti, Chiara Canori, Ilan Shimshoni, Annika Bremhorst

**Affiliations:** 1 University of Haifa, Haifa, Israel; 2 University of Parma, Parma, Italy; 3 Dogs and Science, Zurich, Switzerland; 4 University of Bern, Bern, Switzerland

**Keywords:** Facial landmarks, AnimalFACS, DogFACS, Facial action units, Animal emotion recognition, Movement detection, Computer science, Animal behaviour

## Abstract

Automated analysis of facial expressions is a crucial challenge in the emerging field of animal affective computing. One of the most promising approaches in this context is facial landmarks, which are well-studied for humans and are now being adopted for many non-human species. The scarcity of high-quality, comprehensive datasets is a significant challenge in the field. This paper is the first to present a novel Dog Facial Landmarks in the Wild (DogFLW) dataset containing 3732 images of dogs annotated with facial landmarks and bounding boxes. Our facial landmark scheme has 46 landmarks grounded in canine facial anatomy, the Dog Facial Action Coding System (DogFACS), and informed by existing cross-species landmarking methods. We additionally provide a benchmark for dog facial landmarks detection and demonstrate two case studies for landmark detection models trained on the DogFLW. The first is a pipeline using landmarks for emotion classification from dog facial expressions from video, and the second is the recognition of DogFACS facial action units (variables), which can enhance the DogFACS coding process by reducing the time needed for manual annotation. The DogFLW dataset aims to advance the field of animal affective computing by facilitating the development of more accurate, interpretable, and scalable tools for analysing facial expressions in dogs with broader potential applications in behavioural science, veterinary practice, and animal-human interaction research.

## Introduction

Many animals are capable of producing a wide range of behavioural expressions, such as variations in body posture, gaze direction, and facial expressions^1–4^. These signals provide a continuous flow of information and can play a crucial role in social interactions, allowing individuals to convey intentions, communicate with others, and potentially express internal states^5–7^. However, studying such behaviours in detail remains challenging. Manual behavioural coding remains one of the most time-intensive tasks in animal science. Despite the wealth of potential information carried in an animal’s facial expressions and body movements, decoding this information still relies heavily on manual coding, typically often requiring specialised and trained observers, frame-by-frame inspection, and considerable effort, which is often difficult to scale to larger datasets or across studies^8^. In response to this bottleneck and with the increasing use of computational methods in animal behaviour research, there is growing interest in automating the analysis of behavioural expressions using machine learning and computer vision^9^. This is particularly relevant when studying potentially subtle or short-lived indicators of affective states, such as emotions or pain^10–13^.

Emotions are complex states involving physiological, cognitive, and behavioural components^14^. These states are internal experiences facilitating adaptive response and are thus expressed through observable behaviours, providing critical information about the animal’s well-being and intentions^15,16^. This way, even changes in facial expressions become a key medium, allowing animals to convey/communicate their internal states to others.

Human emotion research has focused on facial expressions for many decades, as they provide a non-invasive, measurable way to recognise emotional states^17^. Similarly, most mammalian species produce facial expressions, and, as in humans, they are assumed to convey information about emotional states, motivations and future intent^4,18^. Therefore, new technologies in facial analysis in animals can lead to new indicators for measuring animal affective states and can revolutionise the way we assess, study, and interpret subjective states, such as stress, emotions, and pain, in domestic species.

Interest in domestic dogs’ behaviour and cognition has increased significantly over the last 30 years^19^. In fact, they also serve as valuable clinical models for numerous human disorders^20^ and are commonly used in research on domestication^21^, attachment^22^, cognition, and more, leading to the multi-disciplinary research field of canine science. In this field, emotions are of increasing interest, addressing both the production of facial expressions of dogs in emotional states, representing potential canine emotion indicators^23^, but also the perception of human emotions by dogs^24^ and of canine emotions by humans^25^. Moreover, due to the remarkable diversity of dog facial expressions^26^, an increasing number of works address objective measurement of dog facial expressions as indicators of emotional states in different contexts^23,27^. Facial expressions have also been studied in the context of understanding dog-human communication (e.g., the impact of dog facial phenotypes on their communication abilities with humans^28^, the effect of facial features on the ability of humans to understand dogs^29^, etc.).

The gold standard for objectively assessing changes in facial expressions in human emotion research is the Facial Action Coding System (FACS)^30^. FACS has recently been adapted for different non-human species, including dogs. The Dog Facial Action Coding System (DogFACS^31^) has been applied in several studies^23,27,28,32,33^ to measure facial changes in dogs objectively. However, using this method for facial expression analysis depends on laborious manual annotation, which also requires extensive specialised human training and certification and may still be prone to at least some level of human error or bias^34^. Some first steps to automating facial movements (action units) detection in dogs were taken by Boneh-Shitrit et al.^35^, but much more data is needed for substantial progress.

Facial landmarks (keypoints/fiducial points) detection^36^ offers an appealing, more lightweight but nonetheless highly objective and precise alternative for automated facial analysis. Landmark locations have been shown to provide crucial insights in the context of face alignment, feature extraction, facial expression recognition, head pose estimation, eye gaze tracking, and other tasks^37–39^. They have also been used for automated facial movement recognition systems^40,41^. An important advantage of landmark-based approaches is their ease of application to video data, producing time series of multiple coordinates, which can then be analyzed^42^, classified^43–45^, or processed for different purposes^46,47^.

Such uses of landmark time-series data are just beginning to be explored in the domain of animal behaviour, primarily for body landmarks (see, e.g.,^48–50^), while facial analysis still remains underexplored. The significant challenges mainly arise from the wide variety of textures, shapes, and morphological structures found across different breeds and species. This is especially true for domesticated animals, such as farm and companion ones, and particularly for dogs, which exhibit the most variability in appearance and morphology among all mammalian species^51^. Moreover, typical datasets with human facial landmarks consist of thousands of images with dozens of landmarks. This abundance of data leads to better model performance, even in challenging scenarios such as occlusions or low-quality images. The animal domain, on the other hand, severely needs landmark-related datasets and benchmarks, as highlighted in Broomé et al.^52^. These are just beginning to be developed for species such as cats^53^, dogs^54^, horses^55^, cattle^56^, and sheep^57^; however, in most cases, the limited number of instances in the training data, the small number of landmarks, as well as lack of justification for their placement in terms of facial muscles, makes these tools inadequate for capturing the subtle facial changes necessary for emotion or pain recognition. Martvel et al.^58^ recently addressed this gap for cat facial analysis by introducing a dataset and a detector model for 48 anatomy-based cat facial landmarks. The detector performed well in several tasks requiring subtle facial analysis, such as breed, cephalic type, and pain recognition^59,60^.

This present study is the first to address these challenges in dog facial analysis, adapting a landmark-based approach. We develop an extensive landmark scheme with 46 facial landmarks grounded in dog facial anatomy and introduce the Dog Facial Landmarks in the Wild (DogFLW) dataset, containing 3732 images of dogs annotated with landmarks and facial bounding boxes. We then utilise different computer vision models to provide a benchmark landmark detection pipeline for the DogFLW. Moreover, we apply and evaluate this pipeline in two case studies related to dog facial analysis from video data: emotional state classification and DogFACS action unit detection.

## Related works

In this section, we examine the current animal facial landmark datasets and their organisation. We then briefly present the Facial Action Coding System and its adaptation to dogs, commonly used for measuring facial appearance changes in dogs, and discuss its benefits and shortcomings. Finally, we discuss works on emotion recognition from animal facial expressions and review studies on the automated detection of facial action units.

### Animal facial landmarks detection

Most of the existing animal datasets mentioned in this section cover a relatively small number of facial landmarks (fewer than 15). For comparison, popular human facial landmark detection datasets have several dozens of landmarks^61–63^. Sufficient for general pose detection, low-dimensional landmark schemes can’t capture subtle facial movements. The advantage of datasets based on those schemes is a large number of instances with a high variation in environment and appearance, since annotating images with fewer landmarks requires less time and effort.

Liu et al.^54^ collected a dataset of 133 dog breeds comprising 8351 dog images, annotated with eight facial landmarks: one landmark per eye, one for the nose, two landmarks on the upper base of ears, two on the ear tips, and one on the forehead. The amount of collected data is excellent, but the suggested landmark scheme doesn’t allow for tracking mouth and tongue movements, which could serve an important role in dog behaviour analysis^64^.

Khan et al.^65^ developed the AnimalWeb dataset, which contains 21,900 images annotated with nine facial landmarks. The dataset includes images of various animals, with around 860 images of dogs. Each image is annotated with two landmarks for each eye, one for the nose and four for the mouth. The proposed scheme doesn’t include ear landmarks at all, which can be used in behaviour analysis as well^66^.

The horse dataset, developed by Pessanha et al.^55^, contains horse head poses divided into three groups: frontal, tilted, and side. Each group has its own landmarks: 54 for frontal, 44 for tilted, and 45 for side views. This approach was chosen to deal with self-occlusions but has two issues: first, the annotator has to decide which landmark scheme to use each time, and second, having three schemes for one animal makes automated landmark detection impractical for real-life applications.

Martvel et al.^58^ created the CatFLW dataset using the cat 48 facial landmark scheme developed by Finka et al.^67^. This scheme is based on the cat’s facial anatomy and enables various applications, such as the classification of animal pain based on the analysis of facial landmarks^60,68^.

Other relevant animal datasets^53,56,57,69–72^ with facial landmarks are listed in Table 1. There are other datasets suitable for face and body detection, as well as recognition of various animal species, but they do not have facial landmark annotations^73–78^.

From the literature on dog facial landmarks, we can conclude that there are no comprehensive datasets covering the nuances of dog facial anatomy and that existing datasets do not share the standard facial landmark scheme, which complicates training computer vision models and following behaviour analysis.

**Table 1:** Comparison of animal facial landmarks datasets.

Dataset	Animal	Size	Facial landmarks
Khan et al.^65^	Various	21,900	9
Zhang et al.^69^	Cat	10,000	9
Liu et al.^54^	Dog	8351	8
Cao et al.^70^	Various	5517	5
Coffman et al.^56^	Cattle	4600	13
Mougeot et al.^71^	Dog	3148	3
Martvel et al.^58^	Cat	2091	48
Sun et al.^53^	Cat	1706	15
Pessanha et al.^55^	Horse (tilted)	952	44
Hewitt et al.^57^	Sheep	850	25
Yang et al.^72^	Sheep	600	8
Pessanha et al.^55^	Horse (frontal)	370	54
Pessanha et al.^55^	Horse (side)	348	45
DogFLW	Dog	3732	46

### Facial action coding systems and DogFACS

The benchmark for objectively assessing changes in facial expressions in human emotion research is the Facial Action Coding System (FACS)^30^. In this system, each facial movement is represented by a variable, either an action unit (AU) or an action descriptor (AD), and is defined based on the underlying muscle activity that produces observable changes in facial appearance. Action units refer to specific, identifiable muscle contractions, while action descriptors are used for movements involving muscle groups or cases where the precise muscular basis is unknown or difficult to isolate. FACS has been extended to animals (AnimalFACS), including non-human primates and domesticated animals, including horses, dogs, and cats^31,79–83^. DogFACS^31^ is developed on a base of dog facial anatomy^84^ and has in total 21 AUs and ADs to track movements in the ear, eye, and mouth region.

Caeiro et al.^32^ applied DogFACS to assess the spontaneous emotional responses of individuals of different breeds in naturalistic settings using videos from the Internet. Unlike that of Caeiro et al., Bremhorst et al.^23^ investigated dogs’ facial expressions of positive anticipation and frustration in a controlled experimental setting, standardising the dog breed (*Labrador Retriever*). Measuring the dogs’ facial expressions using DogFACS, Bremhorst et al. showed, e.g., that the *ears adductor* variable was more frequently observed in the positive condition. In contrast, *blink*, *lips part*, *jaw drop*, *nose lick*, and *ears flattener* were more common in the negative condition. In a subsequent study, Bremhorst et al.^85^ replicated their experiment with a new group of dogs, new controlled settings, and disentangling expressions likely linked to emotions from those of the underlying motivation state.

Boneh-Shitrit et al.^35^ used the dataset and DogFACS codings from Bremhorst et al.^23^ and showed that a machine learning model could classify the emotional state based on manual DogFACS coding with an accuracy of 71%.

Pedretti et al.^27^ further investigated the relation between dogs’ emotional states and facial expressions across different breeds, replicating the experimental approach of Bremhorst et al. but introducing the social factor (a human experimenter). They found that some movements (*ears flattener*, *blink*, and *nose lick*) are more common in the social context of frustration, than in the non-social one.

Sexton et al.^28^ analysed dog facial movements during social interactions with humans in four contexts—non-verbal: without and with eye contact, and verbal: without and with familiar words. The authors found that dogs display various DogFACS action units more with increased communication activity, varying in quantity and diversity and depending on the dogs’ facial colours).

Most studies using DogFACS are conducted manually, so there is room for human bias and error. Slight and fast movements can be challenging to spot in long videos, and it’s not always easy to determine their boundaries and duration. This leads to the need to investigate the automation of FACS variable detection, which we explore in this study using a landmark-based approach.

### Emotional state classification in dogs

There is no consensus on the definition of animal emotions^86,87^. However, they are often characterised as internal states expressed in physiological, cognitive, and behavioural changes^88^. Measuring animal emotions is particularly challenging due to their internal and subjective nature and the lack of a verbal basis for communication^14^. One of the non-invasive ways of doing so is measuring behavioural changes and facial signals, which convey emotional information in most mammals^89–92^. Recently, the number of studies addressing emotion recognition in animals is growing. Broomé et al.^52^ provides a comprehensive survey of more than twenty computer vision-based studies on recognising animal pain and emotional states. Below, we review the most relevant works that focus on dogs.

Boneh-Shitrit et al.^35^ used the dataset collected by Bremhorst et al.^23^ to compare two different approaches to emotional state classification from facial expressions: DogFACS coding-based machine learning model and deep learning model (operating on a single-frame basis). While the latter reached better performance (above $89\%$ accuracy), it is less explainable than the DogFACS one.

Hernandez et al.^93^ created a dataset of 7899 images with dogs in four emotional states: fear, contentment, anxiety, and aggression. Computer vision classification models trained on this dataset show good performance (0.67 *F*1 score), but the quality of images (all images were collected from the Internet using keywords) and reliability of the ground truth labels (authors report fair-to-moderate agreement for all classes) remain questionable.

Franzoni et al.^94^ classified three emotional states in dogs: anger, joy, and neutral, attributing a facial expression to emotion (growl—anger, smile—joy, sleep—neutral). Authors reported $\sim$ $95\%$ accuracy of classification of facial expressions, stating that the created computer vision model “is indeed able to recognise in dog images what humans commonly identify as dog emotions”^94^. One can notice that the authors implied the classification of emotions, although they used the concepts of facial expressions and classified them specifically. We agree that emotional state classification can be based on facial expressions, but it is essential to differentiate between a direct correspondence and a statistical correlation between facial expressions and emotions. In the following paper, Franzoni et al.^95^ used the dataset created by Caeiro et al.^32^, which comprises videos of dogs in states of happiness, positive anticipation, fear, frustration, and relaxation. Using different preprocessing techniques and FACS coding, the authors achieved $62\%$ accuracy in emotional state classification. They also pointed out that the environment could bias deep learning methods for classification in the wild. For instance, a computer vision model might classify a dog on a couch as sleeping not necessarily because of its current activity but because the training data contains a prevalence of sleeping dogs on couches. Franzoni et al. used various techniques to mitigate bias, including facial and body bounding box cropping and segmentation. Their findings indicate that the best classification performance was achieved using facial bounding boxes, which aligns with our face-focused approach.

### AnimalFACS event detection

Another essential task in animal behaviour analysis is movement detection and recognition. Since most animals cannot explicitly translate their emotions and pain verbally, movement detection can be one of the non-invasive ways to translate an animal’s emotional state. Facial movements have their specifics since they can be slight or vague (such as half-blinks or lip corner movements) and are hard to define clearly in real-world data. The abovementioned AnimalFACS describes facial movements using unified schemas for specific animals, allowing different researchers to operate in the same terms, but this approach requires an enormous amount of manual annotation work.

Automated movement detection and recognition is a well-developed field in humans^96–101^. In animals, such automatization is lacking and only beginning to appear.

Morozov et al.^102^ classified six MaqFACS variables in macaques, reporting $81\%$ and $69\%$ accuracy for upper and lower face parts within one individual, $75\%$ and $43\%$ accuracy across individuals of the same species (*Macaca Mulatta*), and $81\%$ and $90\%$ accuracy across breeds (*Macaca Fascicularis*). The studied variables had different frequencies within the dataset used, so the authors performed undersampling of video frames, obtaining 1,213 and 310 images per variable of upper and lower face parts. Additionally, Morozov et al. showed the application of automated movement detection, performing a behaviour analysis on videos with macaques reacting to the appearances of other individuals.

Li^103^ classified nine EquiFACS variables from horse images, with *F*1 score varying from 0.56 to 0.73. Despite the demonstrated potential of classification of horse facial movements from images, the author reports the impossibility of detecting ear movements without temporal information and suggests using “sequence models for horse facial action unit recognition from videos to learn the temporal information of AUs”.

Boneh-Shitrit et al.^35^ detected nine manually coded DogFACS variables, sampling frames from manually annotated videos with *Labrador Retriever* dogs. Performing a “one versus all” classification, the authors achieved detection performance varying from 0.34 to 0.76 *F*1 score, which almost linearly correlates with the number of training samples. Such an approach allows the detection of frequent FACS variables in videos, processing them frame by frame, with a limitation of the deep learning architecture that does not allow transferring motion detectors to other breeds.

When creating automated methods to detect animal movements, it is vital to consider the possible bias and validity of such tools^104^. Various animal appearances and complications related to real-world environments (such as the low resolution of videos with wild animals) create possible errors in movement detection. In the current study, we utilise the same-breed *Ladrador Retriever* dataset^23^ for movement detection, almost mitigating the dog appearance variations, with the same laboratory environment, not introducing noise (occlusions, weather, lightning, etc.) into the data. Such an approach to facial movement detection limits the applicability of the created tool but ensures its validity as much as possible.

## The DogFLW dataset

In the present study, we have created the DogFLW dataset, inspired by existing ones for humans and animals, to promote the development of automated facial landmark detectors in dogs. Next, we present the processes of developing the landmark scheme and data annotation, as well as benchmark results for landmark detection on the DogFLW dataset.

### Dataset

#### Landmark scheme

The 46 facial landmark scheme was developed by experienced dog behaviour experts and active canine science researchers certified in DogFACS. To establish the number of landmarks and each landmark location, the experts worked independently using two different approaches and then converged using expert consensus. The first approach is based on the anatomy of canine facial musculature and the range of possible expressions and facial movements to ensure the connection between landmark positions, possible facial movements, and underlying facial musculature. The second approach was largely inspired by the landmark scheme development for cats^67^, informed by the CatFACS coding system^83^. Analogously, the developed dog landmarks were based on DogFACS^31^.

**Fig. 1:**
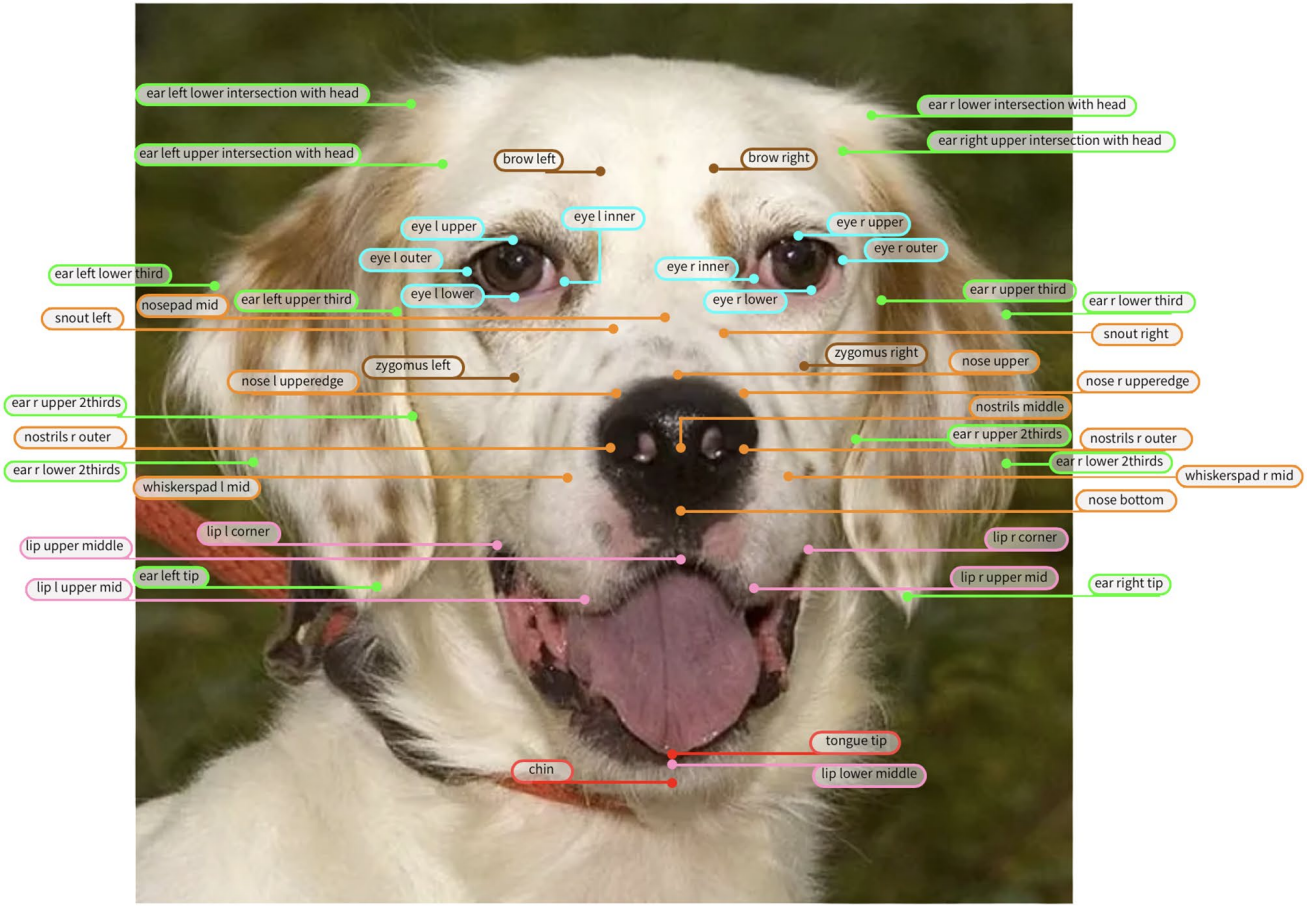
Annotated Dog’s Face. Image of a dog with 46 facial landmarks.

After independently developing the two landmark schemes, both approaches converged. The experts compared the set of landmarks, and those consistent across both approaches were retained while differing landmarks were discussed thoroughly to reach an expert agreement on the precise location. The final landmark scheme is presented in Fig. 1.

Like in Finka et al.^67^, a comprehensive manual was developed detailing landmarks placement and their relevance to facial musculature and action units. This manual serves as a guide for accurately annotating canine facial landmarks, ensuring consistency and reliability in future research and applications, and is available in the Supplementary Materials and by the link https://rb.gy/r6srv9.

#### Annotation

As a source for the DogFLW dataset, we used the Stanford Dog dataset^105^, which contains 20,580 images of 120 dog breeds with bounding boxes.

First, we selected a random subset of images with an equal number of images per breed. Then, we filtered the images according to the following criteria: the image contains a single, mostly visible dog face. Other dogs could be present, but their faces shouldn’t be visible for the unambiguity of detection. Unlike in the CatFLW, we included images with partially occluded faces, estimating the positions of landmarks that were not visible.

**Fig. 2:**
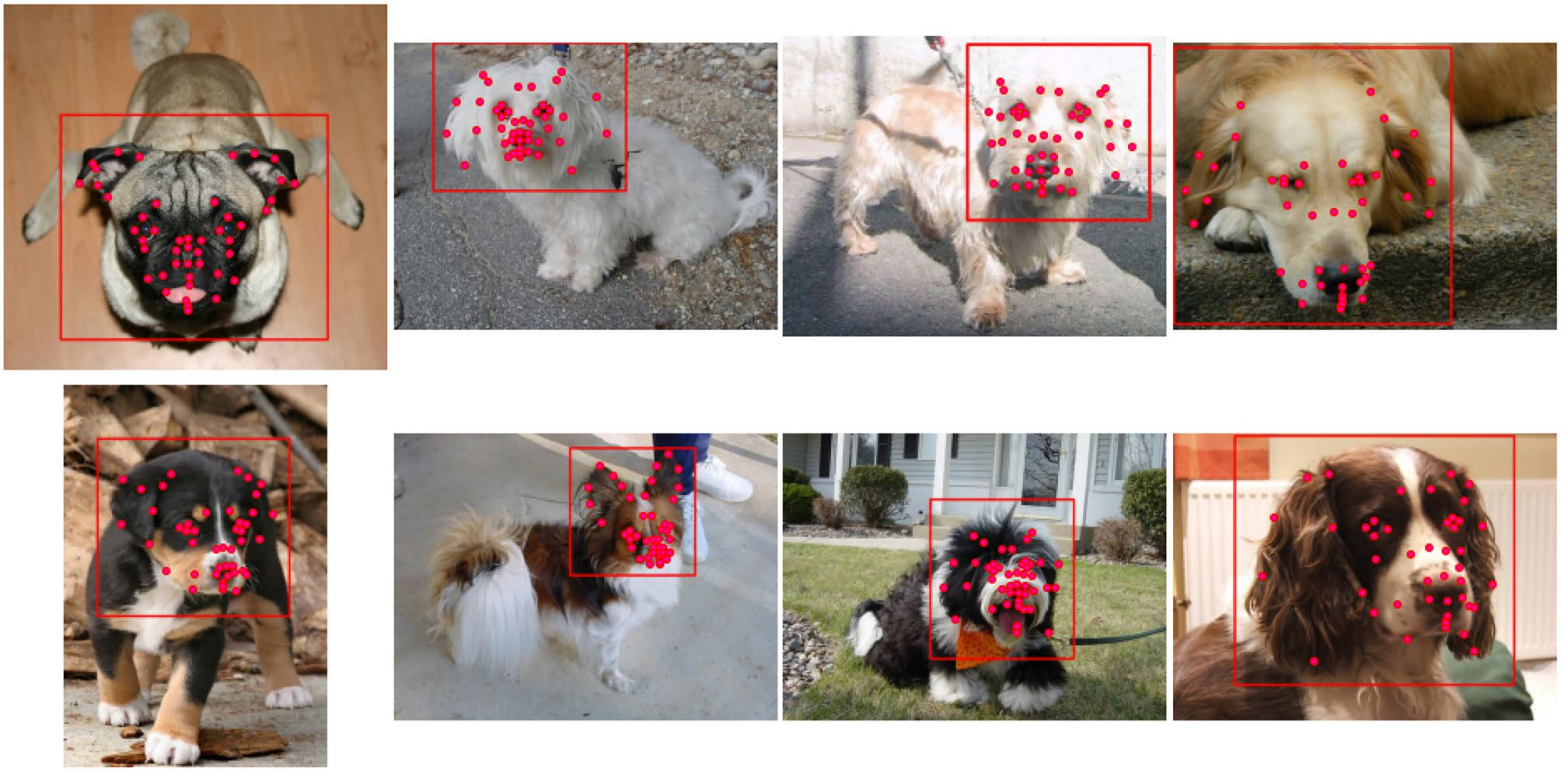
Examples of annotated images from the DogFLW..

The resulting subset contains 16–53 images per breed (31 on average) of all 120 breeds (3732 images total), ranging in size from $100\times 103$ to $1944\times 2592$ pixels. Dogs in filtered images have different sizes, colours, body and head poses, as well as different surroundings and scales. All images are annotated with 46 facial landmarks using the CVAT platform^106^ according to the established scheme. Each image is also annotated with a face bounding box, which encompasses the dog’s entire face, along with approximately 10% of the surrounding space. This margin has proven crucial for training face detection models, as it prevents the cropping of important parts of the dog’s face, such as the tips of the ears or the mouth. Figure 2 shows examples of annotated images from the DogFLW dataset.

The dataset was annotated by an experienced data annotator, then the annotated samples were iteratively reviewed by the last author, a certified DogFACS coder, until annotation saturation was reached, e.g., no significant corrections were needed.

To make the annotation process more efficient, we followed the active learning AI-assisted annotation process^58,107,108^, dividing the dataset into ten batches and annotating training data using predictions of a machine learning model that is gradually being retrained on previously corrected data. More information on the active learning paradigm can be found in the study^109^.

### Landmark detection benchmark

The dataset was divided into the train (3252 images) and test (480 images) sets for evaluation. The test set contains an equal number of images of all 120 breeds present in the dataset. In the preprocessing stage, we cropped all the faces by their bounding boxes to provide fair metrics for all models.

We performed landmark detection on a Supermicro 5039AD-I workstation with a single Intel Core i7-7800X CPU (6 cores, 3.5 GHz, 8.25 M cache, LGA2066), 64GB of RAM, a 500GB SSD, and an NVIDIA GP102GL GPU.

#### Metrics

We use Normalised Mean Error ($NME_{iod}$) that preserves the relativity of the error regardless of the size of the image or the scale of the face on it. It is commonly utilised in landmark detection^110–112^, and uses MAE as the basis and inter-ocular distance (distance between the outer corners of the two eyes, IOD) for normalisation:

$$\begin{aligned} NME_{iod} = \frac{1}{M \cdot N} \sum _{i=1}^{N} \sum _{j=1}^{M} \frac{\left\| {x_i}^j - {x'_i}^j \right\| _1}{iod_i}, \end{aligned}$$

where *M* is the number of landmarks in the image, *N* is the number of images in the dataset, ${x_i}^j$ and ${x'_i}^j$ — the coordinates of the predicted and ground truth landmark, respectively.

#### Baselines

To measure the performance on the DogFLW, we have selected the following models: Ensemble Landmark Detector (ELD)^58^, DeepPoseKit (DPK)^108^, DeepLabCut (DLC)^113–115^ and Stacked Hourglass^116^. Those models are widely used in the animal field and are usually viewed as the default for the animal body/facial landmark detection^52,117,118^. The training process for all the models except ELD was performed using the DeepPoseKit platform^108^. All models were trained for 300 epochs with a batch size of 16, mean squared error (MSE) loss, the ADAM optimiser, and optimal parameters for each model (indicated in the corresponding papers).

#### Preprocessing

We randomly applied different augmentations (rotation, colour balance adjustment, brightness and contrast modification, sharpness alteration, application of random blur masks, and addition of random noise) to the training data, doubling the size of the training set.

#### Results

The results of landmark detection with different models and backbones on the DogFLW are shown in Table 2.

**Table 2:** Comparison of landmark detection error on the DogFLW dataset using different detection models.

Model	Backbone	NME
DLC^113^	ResNet101	9.94
DLC^115^	MobileNetV2	7.37
DPK^108^	Stacked DenseNet	7.10
Stacked Hourglass^116^	Hourglass	6.87
DLC^114^	Xception	6.71
DLC^113^	Densenet121	6.70
ELD^58^	EfficientNetV2S	6.52

During the experiments, it was noticed that the accuracy of detecting ear landmarks is significantly lower compared to other facial parts across all models. This is likely because of the varying shapes and lengths of ears among different breeds, which may include ear types and positions not previously encountered in the training set, especially for floppy or half-floppy ear types. To test our hypothesis, we divided the data into three subsets: one with erect ears (pointy), another with hanging ears (floppy), and the third with other types (half-floppy). When dividing, we were guided by the average breed ear type in a relaxed state, obtaining a ratio of 31:50:19 for the training set and 29:52:19 for the test set. Despite the dataset’s predominance of breeds with floppy ear types, Table 3 shows that the accuracy of the ELD model landmark detection on a test set for such dogs is less than for dogs with erect and half-floppy ears.

**Table 3:** Normalised mean error of landmark detection of the ELD model on the test set for dogs with different ear types.

Ear type	% in train set	NME total	NME ears only
Pointy	31	5.31	9.79
Floppy	50	7.18	15.28
Half-floppy	19	6.56	12.77

**Fig. 3:**
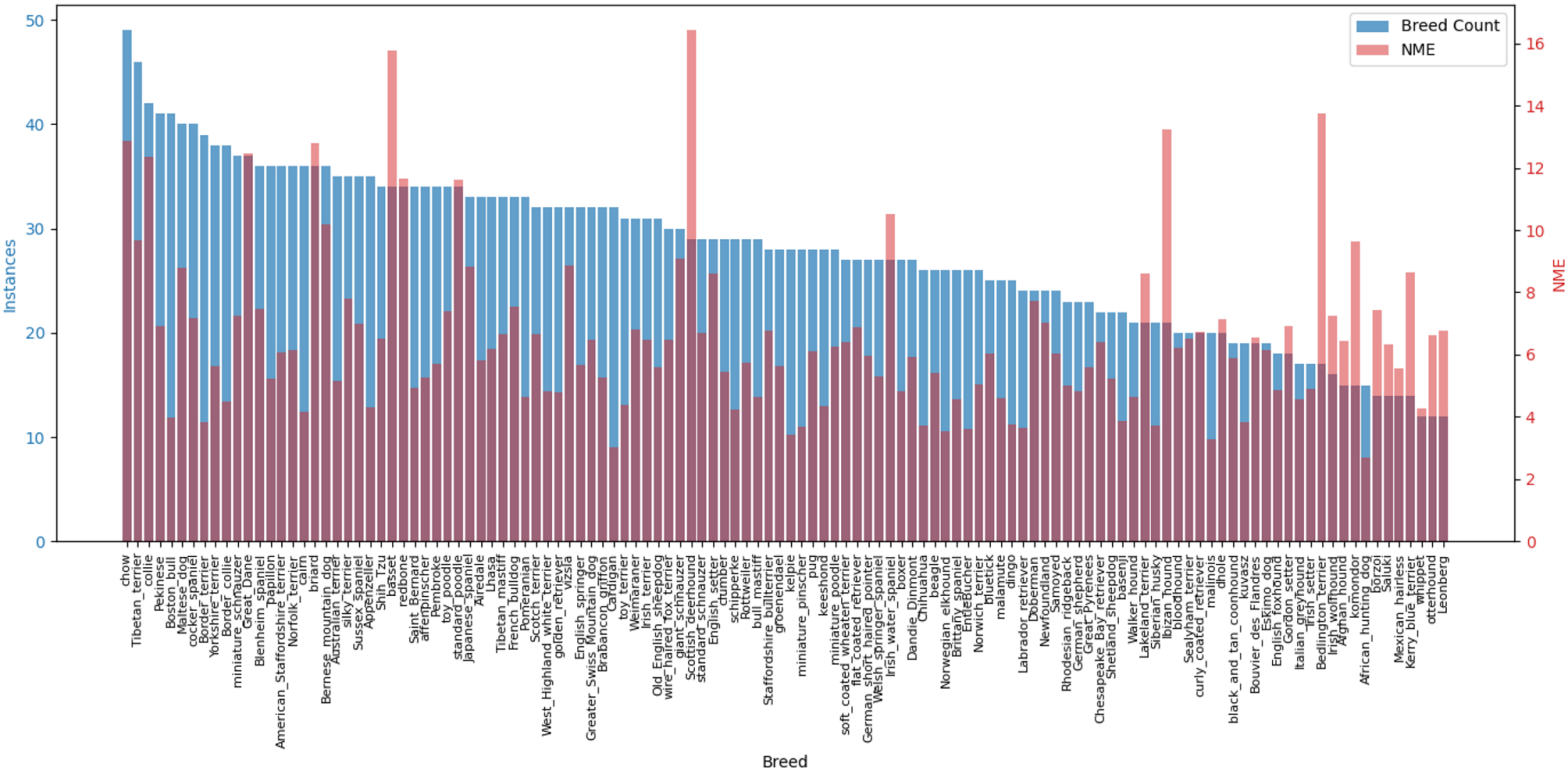
The distribution of images in the training set for each dog breed (blue) and the ELD’s detection errors for the same breeds in the test set (red).

Due to the uneven presence of breeds in the training set, the accuracy of landmark detection for rare breeds may be lower than for frequent breeds. However, it would be incorrect to attribute detection errors solely to the number of samples. The breed itself plays a significant role in detection accuracy, as many breeds have distinct fur length, texture, and facial anatomy that influence the results. Figure 3 shows the image distribution of dogs of each breed in the training set and the ELD’s detection error for the same breeds in the test set. Based on the distribution, it can be observed that breeds with long fur covering facial features or extending on ears are the most difficult to detect accurately (*Irish Water Spaniel, Briard, Standard Poodle, Bedlington Terrier, Scottish Deerhound, Komondor, Kerry Blue Terrier*). Moreover, we previously demonstrated that detection has a high error on dogs with long, floppy ears, resulting in low accuracy in detecting facial landmarks for breeds with such ears (*Basset, Redbone*). It is also worth noting that some breeds have non-obvious detection results. In some cases, this could be explained by a significant variation in the position of the ears (*Ibizan Hound, Collie, Great Dane*) or by their being obscured (*Chow*), which can cause a significant detection error. It can also be seen that breeds with short and smooth facial fur, such as *Cardigan, Kelpie, Miniature Pinscher, Dingo, Chihuahua,* and *Malinois*, tend to have the lowest average detection error, as their facial features are more clearly distinguishable.

We additionally evaluated the generalisability of the ELD model to assess how it performs on unseen breeds. For that, we conducted an additional experiment using a 2 × 2 design that systematically varied two key morphological features in canine faces: ear type (erect vs floppy) and snout length (short vs normal/long). This resulted in four distinct morphological combinations. For each combination, we selected one representative breed and excluded all images of that breed from the training set, using it only for testing. The selected breeds were: *Labrador Retriever* (normal/long snout, floppy ears), *Boxer* (short snout, floppy ears), *French Bulldog* (short snout, erect ears), and *German Shepherd* (normal/long snout, erect ears). Accordingly, we generated four subsets, excluding all images of dogs of the selected specific breeds from the training set (one per subset: *Labrador Retriever*: 26 images, *Boxer*: 30 images, *French Bulldog*: 39 images, *German Shepherd*: 24 images). Then, we trained four models on the resulting subsets and evaluated them on the original test set. The results, provided in Table 4, demonstrate that the exclusion of one breed from the training data almost does not impact the model’s performance in general, but reduces the performance on that specific breed in the test set. This could be explained by the fact that excluded breeds take about $1\%$ of the training data, so the general performance doesn’t drop significantly. However, the varying decrease in performance between excluded breeds could be explained by the presence or absence of breeds with similar morphology in the training set.

**Table 4:** The normalised mean error of landmark detection for the ELD model, trained on different subsets from DogFLW, is evaluated on the test set. In the case of “included,” all breeds were present in the training set. Conversely, in the “excluded” case, the selected breed was absent from the training set. The error is reported for both the full test set and for the selected breed within the test set.

Breed	Included		Excluded	
	Full test	Breed	Full test	Breed
*Labrador Retriever*	6.52	3.64	6.52	3.75
*Boxer*	6.52	4.85	6.52	5.54
*French Bulldog*	6.52	7.69	6.51	7.78
*German Shepherd*	6.52	4.7	6.52	4.72

## Methods

To investigate the applications of the landmark-based approach, we use the dataset from Bremhorst et al.^23^. The dataset contains recordings of 29 *Labrador Retriever* dogs (248 videos total) in a controlled laboratory setting, inducing two emotional states: positive (anticipation of a food reward) and negative (frustration due to the reward’s inaccessibility). In addition to the labels for positive/negative emotions, the videos were coded using DogFACS.

The DogFLW dataset provides an excellent opportunity for testing the trained landmark detector for complex, subtle video analysis tasks related to dog facial behaviour. The specific tasks we chose are (i) emotion recognition (positive/negative) and (ii) DogFACS event (variable) detection.

We used the Google Colab cloud service (https://colab.research.google.com) with the NVIDIA TESLA V100 GPU to train and evaluate the video classification and movement detection models. All models were trained and evaluated using TensorFlow 2.17^119^, pandas 2.2.3^120^, and NumPy 2.1.0^121^.

### Emotion recognition

**Fig. 4:**
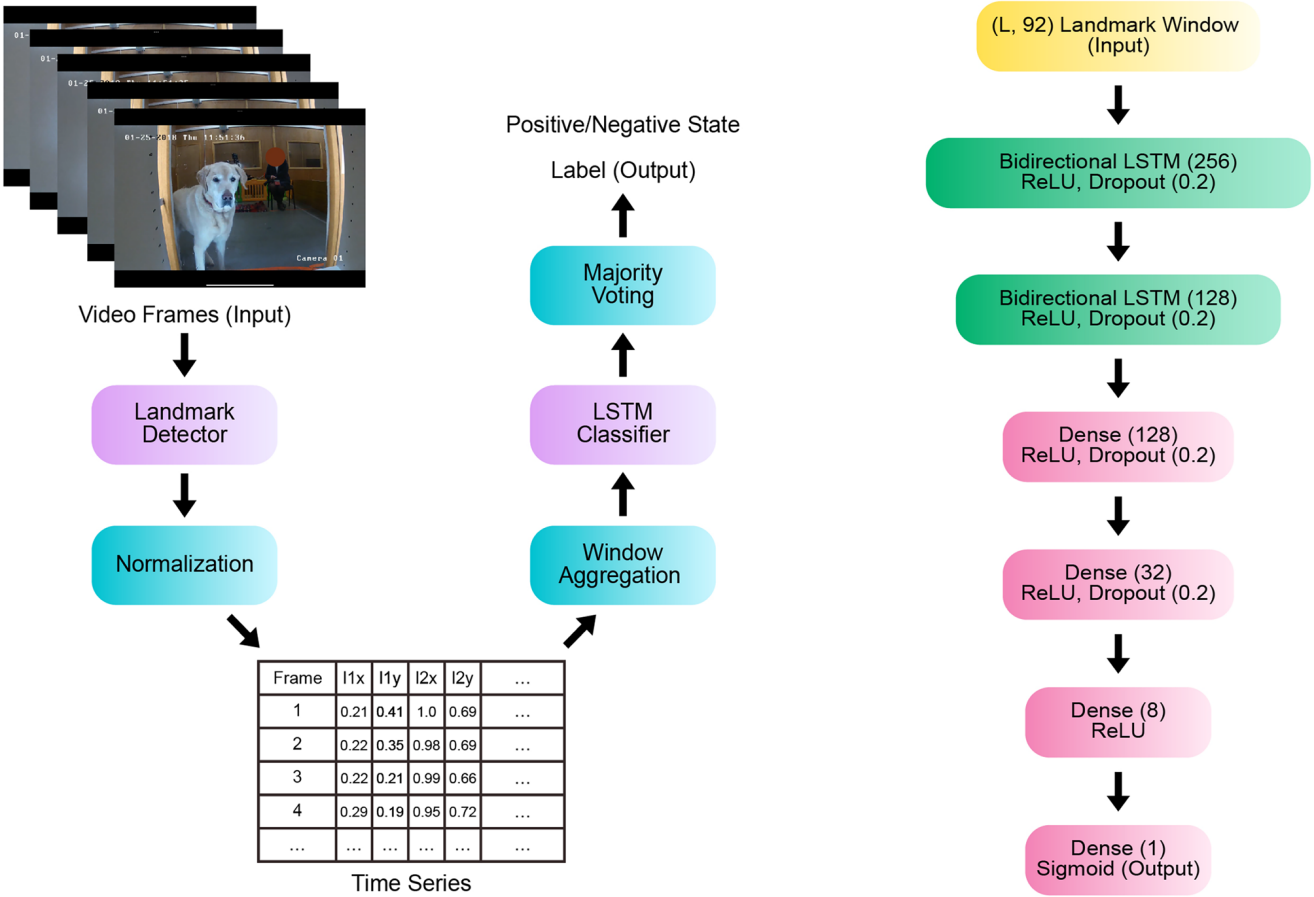
Video Classification Pipeline (left). In each video frame, 46 facial landmarks are detected, which are then normalised and organised into time series. These series are processed using a sliding window and an LSTM classifier model. For each window, the vote is saved, and the decision for the entire series is made based on majority voting. LSTM Classifier Architecture (right). Two bidirectional LSTM layers are followed by four narrowing Dense layers. The model outputs a probability of a landmark window belonging to a certain class.

The first classification task is defined as follows: given a video of a dog, classify whether it is in a positive or negative emotional state. To this end, we integrated facial landmark prediction as a middle step and use the obtained time series for classification. The pipeline is described in Fig. 4(left).

#### Landmark detection

We processed all 248 videos from the dataset with the Ensemble Landmark Detector (ELD)^58^, predicting landmarks on each frame. We chose the ELD model for its performance, introducing two minor changes to the architecture. First, as a face detector, we use the custom-trained YOLOv8^122^ model. This choice is crucial for video processing since the original model’s architecture implies the presence of the animal’s face on the input image. In videos, it is not always true due to animals’ movements, head rotations, obscurity, etc. Moreover, we trained a fully connected verification model that takes landmarks as input and outputs a probability score. This score could be interpreted as the model’s certainty regarding the quality of the obtained landmarks. This model consists of three fully connected layers (128, 32, 1) and was trained on the DogFLW landmarks as positive examples and landmarks predicted on random images as negative examples.

The resulting landmarks from all videos were normalised and saved as a time series database. Each landmark row has 46 landmark coordinate pairs, as well as the verification model’s confidence. In some frames, landmarks were not detected (the dog was turned away/heavily obscured, etc.), so we assigned zero values to landmark coordinates and zero confidence for the whole frame.

#### Preprocessing

We intentionally left frames with no landmarks detected in the obtained time series without interpolation or filling. No detected landmarks in almost all cases means that the dog is heavily rotated/obscured, and its face is mostly not visible. Of course, the human annotator can still draw some information from such frames, but it’s impossible for the landmark-based model. Using an interpolation, we introduce artificial landmark sequences, which are not related to the real video in any way and may contain false signals. When doing window aggregation from time series, we skipped windows with one or more empty frames to ensure the model was trained on informative data.

We split the time series data into training and test sets. The training set was processed with a sliding window of size (*L*, 92) with a step of $S=1$. We additionally experimented with sparse windows, which have the same length as regular ones but capture more temporal information by skipping rows. The sparse window could be considered a standard sliding window for reduced fps. For example, a standard window with $L=5$ and 25 fps captures 0.2 seconds, and a sparse window with the same length and 5 fps (3 rows skipped each time) captures 1 second. Such an approach allows for the investigation of the impact of the temporal dimension without increasing computational resources. The resulting aggregated windows were divided into two classes based on the dog’s state in the original video, and then balanced by downsampling. We used sparse windows with $L=5$ and 14 fps for the current experiment (chosen empirically through a grid search), resulting in 11,922 windows total.

#### Metrics

To measure the classification performance, we implemented a majority voting mechanism over the sliding window predictions: the model predicts a class for each window, and then the dominant class is assigned to each video as a label. Obtaining the label for each video, we measured accuracy, precision, recall, and *F*1 score of the classification.

Since the dataset consists of 29 dogs and the same dog being in both training and test sets could introduce data leaks, we used the leave-one-animal-out cross-validation method, commonly applied to such tasks^52^.

#### Baseline

To classify the landmark windows, we implemented an LSTM-based model, which consists of two bidirectional LSTM layers^123^, followed by four Dense layers (see Fig. 4(right)). Taking as input a window of size (*L*, 92), it outputs a probability of this input window belonging to one of the two classes: positive or negative. The model was trained for 300 epochs with a batch size of 16, MSE loss, and an ADAM optimiser.

### DogFACS event detection

The second task we investigated was the following: given a video, detect movements (events) in the form of various DogFACS action units (and descriptors), and determine the start and end time of the event. In addition to investigating detection performance, we measure its usefulness for computer-assisted DogFACS annotation in a pilot experiment with a DogFACS-certified coder.

The dataset of Bremhorst et al. provides an excellent opportunity in this context as it is DogFACS-coded (start/end time for each of the DogFACS variables). Since DogFACS elements have two types (action unit and action descriptor), they are usually referred to as DogFACS variables. We refer to them in our context as “DogFACS events” for simplicity.

Our approach to this type of event detection is to treat meaningful events as time series anomalies. This is justified by the fact that facial movements are usually localised and could be separated from the animal’s general movement. That means if we accept the relaxed facial expression as normal, raising brows, opening the mouth, and other facial movements disrupt this normal state, but in a “localised” way. By utilising facial landmarks (which change their coordinates as the face changes its expression), it is possible to translate the occurring movement into coordinate time series, where classical time series analysis could be applied to detect this movement. For instance, if the dog is moving within the frame, in most cases, we can assume that the trajectory of each facial landmark follows the same direction and has a similar pattern to others. If a relative facial movement occurs, the trajectories of several landmarks change within the global pattern, and this variation could be potentially seen in the coordinate plots.

**Fig. 5:**
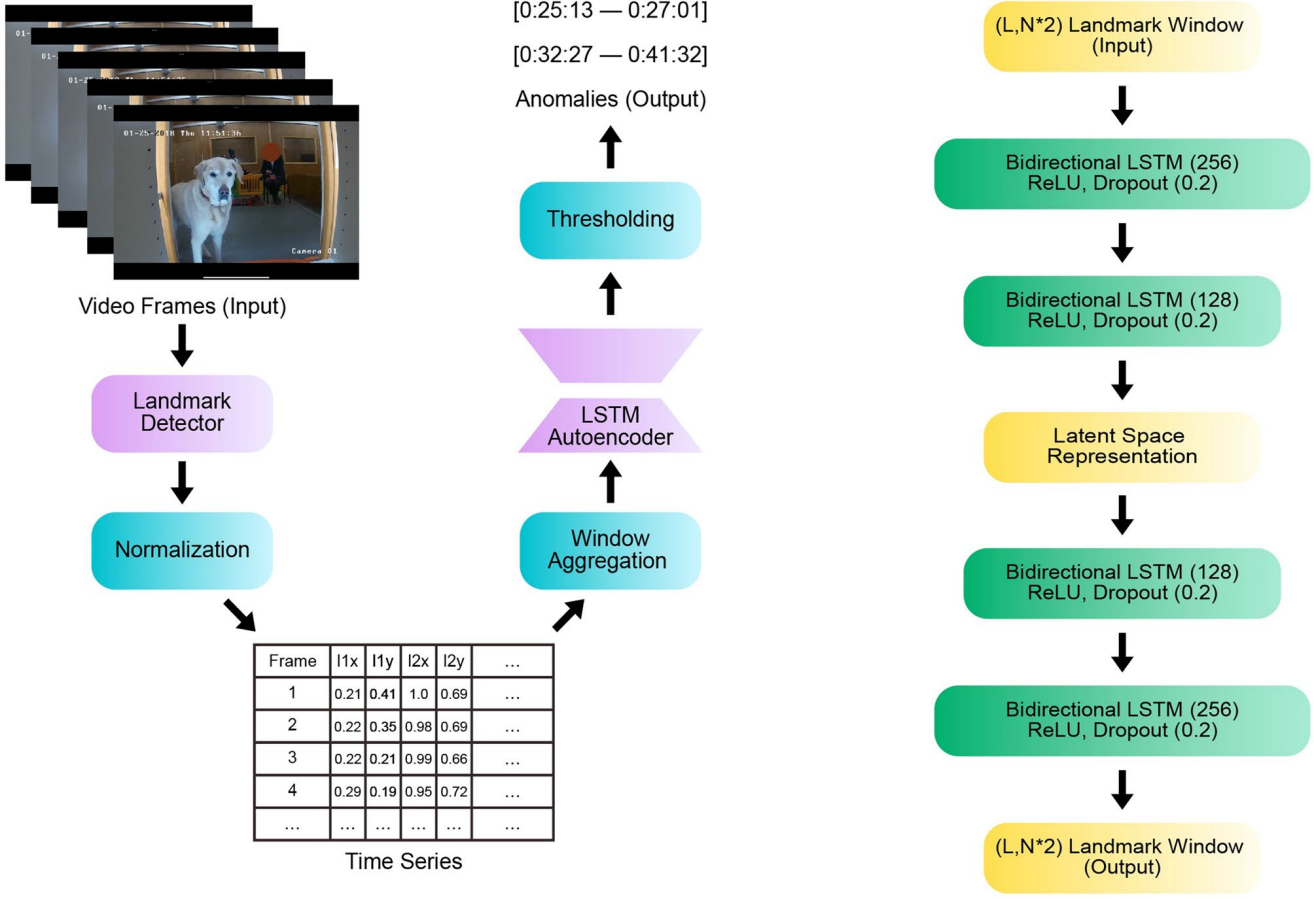
Video Anomaly Detection Pipeline (left). In each video frame, 46 facial landmarks are detected, normalised, and organised into time series. These series are then processed using a sliding window and an LSTM autoencoder model. The autoencoder reconstructs the output sequence and then compares it to the input sequence. An anomaly is detected if the error between the two sequences exceeds a certain threshold. LSTM Autoencoder Architecture (right). The encoder and decoder consist of two bidirectional LSTM layers with ReLU activation and a recurrent dropout.

Therefore, we treat time series obtained from DogFACS events (such as blinking or licking) as anomalies, given that such events are comparably short (typically less than a second) and rarely occur in this dataset. To effectively detect rare movements in videos, we implement an LSTM autoencoder model, which operates on multivariate facial landmark time series obtained from the videos with a landmark detection model (see Fig. 5(left)).

#### Dataset

Bremhorst et al. coded various DogFACS variables, as well as head movements for each video, such as *tongue show*, *head turn left/right* or *ears rotator*, totalling more than 30 instances. Variables are coded every 0.2 seconds, creating 5-frame sections with some movement. Some variables have smaller durations but are still coded as 5-frame sections.

All facial variables are categorised according to DogFACS guidelines: Upper Face Action Units, Lower Face Action Units, Action Descriptors, and Ear Action Descriptors. Other coded variables could be found in the original data and comprise body movements (*body shake*), activities (*panting*), or gaze/head directions (*eyes up* or *head turn right*). There are two main reasons for selecting variables belonging only to the facial categories and discarding others. First, since we want to utilise facial landmarks, we must limit ourselves to only facial movements. Second, we want to treat events as anomalies rather than trends. By that, we mean that the events such as *head tilt left* imply not the movement but the state and, most of the time, last for several continuous sections. A future research direction would be to use a landmark-based approach for gaze/head direction classification.

Each category has several variables, each with a specific number of occurrences in the dataset. We excluded variables with less than 20 occurrences to ensure meaningful statistical analysis across all variables. Sections without any variables in a specific category are labelled as *None*. For each variable, we also provide intercoder reliability (ICR), as reported by Bremhorst et al. Table 5 presents the final list of variables used. The data is collected in such a way that, despite the anomalous events being coded as 5-frame sections, some of them have a point-wise nature, occurring rarely and lasting no more than one sequence (*blink*, *upper lip raiser*). Later, we will refer to one-sequence anomalies as point anomalies.

**Table 5:** DogFACS events (variables) from^23^ used in the current study.

Category	DogsFACS variable	Sections	ICR
Upper face AU	None	3370	–
	Inner Brow Raiser (AU101)	420	0.75
	Blink (AU145)	97	0.8
Lower face AU	None	3542	–
	Jaw Drop (AU26)	125	0.9
	Nose Wrinkler & Upper Lip Raiser (AU109+110)	110	0.5
	Lip Corner Puller (AU12)	51	1.0
	Lower Lip Depressor (AU116)	26	1.0
	Upper Lip Raiser (AU110)	20	0.19
Action descriptors	None	3779	–
	Tongue Show (AD19)	73	1.0
	Nose Lick (AD137)	29	1.0
Ear action descriptors	None	1862	–
	Ears Flattener (EAD103)	1490	0.92
	Ears Forward (EAD101)	410	0.52
	Ears Adductor (EAD102)	83	0.78

#### Landmark detection

In the current experiment, we used the landmark series obtained in the previous video classification experiment.

#### Preprocessing

For each variable *M*, we divided the normalised landmark series into learning and evaluation. To correctly measure the performance, we ensured each evaluation sequence contained at least one anomaly section of the chosen variable. Then, for the learning part, we sliced the time series into windows of size $L=15$ (0.6 sec., three variable segment sizes) with a step of $S=1$ (0.04 sec.). Discarding windows that contain zero-confidence rows (landmarks are undefined), we obtained 12,043 normal and anomaly windows of shape (*L*, 92). An anomaly window contains a full section with a specific variable *M*, while a normal window may include other or no variables. Then, we divided normal windows into train/validation/test in 80:15:5 proportion, leaving all anomaly windows for evaluation.

Each variable category focuses on a specific part of the animal’s face: Upper Face AU for the eyes, Lower Face AU and Action Descriptors for the nose and mouth, and Ear Action Descriptors for the ears. This approach allows us to select only *N* landmarks corresponding to the chosen region, reducing the window’s dimensionality to $(L, N \cdot 2)$.

#### Metrics

Following the research on anomaly detection in time series^124,125^, we used unweighted precision, recall, and *F*1 scores for evaluation of predicted anomaly sequences. When detecting anomalies in satellite signals or electrocardiograms, it’s crucial to minimise false positive detections as they can result in high time and resource costs^126^. In the case of detecting DogFACS variables, the manual annotation process is time-consuming and requires an expert to inspect every frame. Even a small reduction in annotation time would be beneficial, but missing even one variable (a false negative prediction) could lead to inaccurate statistics for the entire video. This is why we consider precision and recall equally important in the current experiment.

To determine introduced metrics, we define the confusion matrix components as follows. If a known anomalous section overlaps any predicted anomaly sequences, a true positive (*TP*) is recorded. If a known anomalous section does not overlap any predicted anomaly sequences, a false negative (*FN*) is recorded. If a predicted sequence does not overlap any labelled anomalous section, a false positive (*FP*) is recorded.

#### Baseline

As can be seen from Table 5, some variables are present in less than 1% of the data, making supervised detection extremely challenging. In^35^, Boneh-Shitrit et al. chose nine variables for supervised image classification, of which only four belong to anomalies, as we define them, while the rest could be classified as trends. It can be seen from the classification results in the original study that only variables with a significant presence in the training data have considerable detection accuracy.

To mitigate the lack of anomaly sections in the training data, we used a variation of an LSTM autoencoder model^127,128^. The autoencoder model was trained on normal data, learning a latent space representation for the input without an anomaly *M* (others could be present) and restoring the input sequence. The difference between input and output landmark sequences is generally small when no anomalies of a particular type are present. However, when the autoencoder encounters anomalous data with an unseen variable *M*, it cannot restore it accurately, leading to a high difference between input and output windows. By measuring the reconstruction error and selecting the threshold, it is possible to determine if the chosen window contains an anomalous variable *M*. Figure 5(right) shows the model’s architecture.

Since we wanted to differentiate variables within each category and the autoencoder model’s architecture implies only binary classification (normal/anomaly), we trained a separate model for each variable *M*. Each model was trained for 200 epochs with a batch size of 16, MSE loss, and an ADAM optimiser.

#### Postprocessing

After obtaining the mean absolute error (MAE) between predicted and input windows for each channel in the landmarks window, the anomaly score was smoothed by the exponential weighted moving average function^129^, and a threshold was applied, resulting in anomaly instances. After that, continuous anomaly instances were merged to create anomalous sequences. We additionally implemented anomaly pruning ($p = 0.13$)^124^ and edge masking with a minimum anomaly score^125^ to reduce the amount of false positive detections.

To determine if a specific error value was considered an anomaly, we analysed the error distribution from the training set to establish a threshold for each landmark coordinate in the evaluation set. We set the threshold using the criterion of $3\sigma$ (empirical value). This was mainly done to adjust the precision/recall balance based on the DogFACS variable under consideration and from the assumption that anomaly data errors land as outliers in the non-anomaly error distribution.

## Results

### Emotion recognition

We compared our pipeline’s performance with the classification results on the same dataset of Boneh-Shitrit et al.^35^, who used DogFACS-based and deep learning approaches. The DogFACS-based approach was performed using manual codings for 39 variables done by Bremhorst et al.^23^ and using 11 automatically detected DogFACS variables. The deep learning approach was performed using ResNet50^130^ and Vision Transformer (ViT)^131^ backbones over the dog facial images cropped from video frames. It can be seen from Table 6 that the deep learning approach outperforms all other classification methods, while the performance of our landmark-based approach is roughly similar to the DogFACS variable-based approaches.

**Table 6:** Accuracy, precision, recall, and *F*1 score comparison using different approaches for the video classification task.

Method	Acc.	Pr.	Rec.	F1
DogFACS (automated)	0.66	0.64	0.67	0.66
DogFACS (manual)	0.72	0.71	0.71	0.71
Deep learning	0.89	0.89	0.89	0.89
Landmarks (ours)	0.76	0.76	0.60	0.67

Despite outperforming other approaches, the deep learning method relies on appearance and, therefore, lacks scalability. For instance, the computer vision model trained on the specific appearance (breed in this case) could perform worse on other appearances, as it was demonstrated for humans^132,133^. The landmark-based approach, on the other hand, operates with numerical data, which contains no information on fur colouration, length, etc., but contains important morphological details, such as distance between lips, blink ratio, etc. This allows us to theorise that even if the landmark-based approach performs less accurately in an emotional state classification task on the particular dataset, it may perform on the same level on other datasets, in contrast to the deep learning approach, which could potentially struggle with breed transfer. Due to the lack of annotated data to test this hypothesis, the study of this issue is planned in our future work.

### DogFACS event detection

We conducted experiments on the evaluation time series separately for each DogFACS variable. The anomaly detection results are shown in Table 7. We also include the percentage of anomaly sections for each variable in the evaluation set.

**Table 7:** Precision, recall, and F1 scores for the DogFACS variables detection.

Variable	Precision	Recall	F1	% Anomalies
Blink	0.240	0.618	0.345	9.62
Inner brow raiser	0.193	0.410	0.263	12.7
Jaw drop	0.114	0.390	0.176	10.16
Nose wrinkler & upper lip raiser	0.221	0.692	0.335	9.18
Lip corner puller	0.333	0.636	0.438	8.15
Lower lip depressor	0.204	0.454	0.281	6.3
Upper lip raiser	0.098	0.555	0.167	6.31
Tongue show	0.222	0.600	0.324	8.44
Nose lick	0.166	0.733	0.270	6.4
Ears flattener	0.278	0.201	0.233	58.78
Ears forward	0.437	0.380	0.407	50.92
Ears adductor	0.189	0.478	0.271	25.69
Average	0.224	0.512	0.292	

The results show that trend anomalies (continuous series of sequences with a high ratio of occurrence, like *ears flattener* or *ears forward*) tend to have lower recall, which means that the model interprets such sequences as normal ones, not being able to distinguish trends, which is a common issue for the anomaly detection models^134^.

As Wong et al.^125^ note, anomalies at the sequence’s beginning can reduce the anomaly detection accuracy. We report a high rate of such variables in the current dataset (54% of videos have a variable present in the first 0.2 seconds of the video, so we assume they were cropped that way), as well as a number of potentially informative frames with no landmarks detected (18% across all videos), which is a limitation of a landmark-based approach.

## Pilot study for computer-assisted DogFACS coding

In the pilot experiment, we developed a prototype for computer-assisted DogFACS coding based on the trained event detector. Although the detector’s accuracy at this stage is insufficient to perform fully automated DogFACS coding, the idea here is to enhance the human coder with suggestions of potentially meaningful time periods representing possible events, measuring the time saved by this enhancement compared to fully manual annotation.

### Video processing

**Fig. 6:**
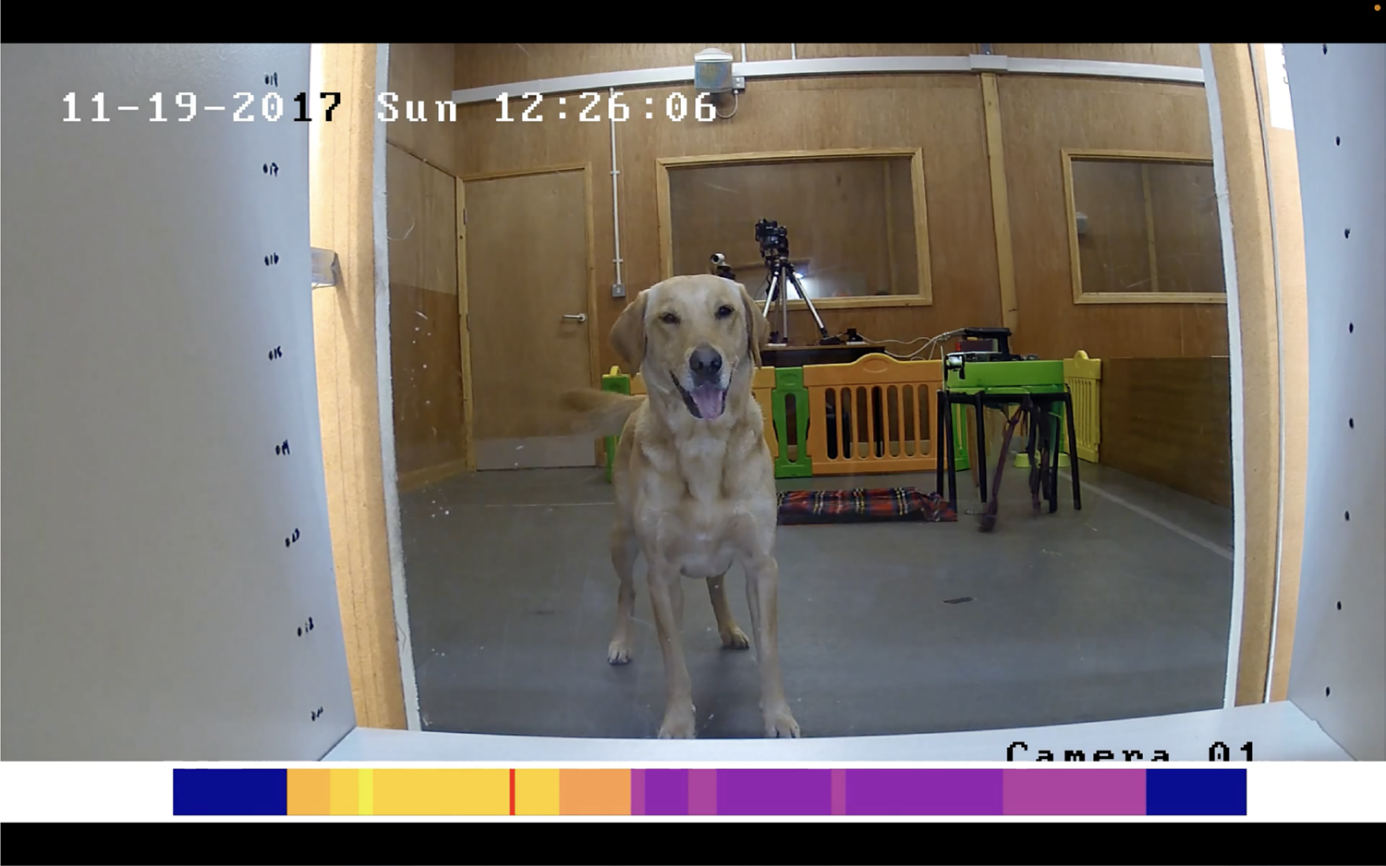
The frame from the processed video. The bar at the bottom displays the intensity of events (blue—the low number of events, yellow—the high number of events) in the current frame, highlighted by the moving slider (red).

In the developed prototype, a colored bar is displayed on top of the processed video, showing the intensity of the aggregated events: blue—lower chance of event, yellow—higher chance of event, with a moving slider (red), indicating the current frame on the intensity plot (see Fig. 6). We decided to merge all DogFACS events into one aggregated bar to reduce the cognitive load for the annotator.

### Experiment

The DogFACS-certified experienced annotator expert coded 20 previously unseen videos from the dataset. Ten videos were manually annotated, and the other ten were annotated using our prototype enhancement. The raw and enhanced videos were displayed randomly to the annotator to avoid bias. The Behavioural Observation Research Interactive Software (BORIS) annotation tool^135^ (version 9.3.1) was used for the coding process, which is common in such annotation tasks^32,81,136^.

Since each video has a different number of DogFACS variables (from one to ten, with 4.2 on average) with different durations, we measured the average time spent on annotating one variable (both start and end) by dividing the total annotation time by the number of annotated variables.

### Results

 A $41\%$ reduction in mean and $26\%$ in median annotation time per DogFACS variable was observed for all annotated videos. This result from our pilot is encouraging, and further work is needed to investigate the best way to enhance DogFACS coding process to reduce annotation time and improve annotation quality. We plan to do so with more datasets, larger annotator teams (also measuring agreement), and further prototype improvement.

## Discussion

This paper introduces the Dog Facial Landmarks in the Wild (DogFLW) dataset, the first large-scale, breed-diverse dataset of 3732 annotated dog images using a 46-point DogFACS-based facial landmark scheme. We provide a benchmark for landmark detection across multiple models and demonstrate the utility of this approach in two applied tasks: emotion recognition from video using landmark time series and the detection of DogFACS facial action units as rare events in time series. These results highlight the feasibility of using a landmark-based approach for fine-grained dog facial behaviour analysis, offering a scalable and explainable alternative to deep learning pipelines.

As expected, the detectors had the most difficulties with ear landmarks due to significant variance in dog ear shapes, which can be improved by enriching the dataset with more samples with diverse ear types. A similar strategy can be taken to improve the detector’s performance on certain breeds that were less represented or had complex morphological features. Additionally, ear-aware augmentation techniques, such as simulating occlusions, varying fur texture, or modifying ear orientation (i.e. through semantic style transfer^137^), may further enhance the model’s generalisation, particularly for dogs with floppy ears or long fur.

We have demonstrated the usefulness of landmark detection for two “proof of concept” tasks requiring a fine-grained and subtle analysis of dog facial behaviour using a dataset from Bremhorst et al.^23^ of *Labrador Retriever* dogs only. The first task is a classification of emotional state from videos using landmark time series as an intermediate step, reaching 76% accuracy. While deep learning models presented in Boneh-Shitrit et al.^35^ outperform our pipeline, the landmark-based approach has the potential benefit of better scalability and explainability while also capturing the temporal dimension (while the deep learning approaches operate on a single-frame basis). We plan to explore this topic more in future work.

The second task we explored as an application of our landmark detector is DogFACS variable recognition. Utilising a landmark-based approach, we were able to detect rarely occurring facial movements, almost non-existent in the dataset, such as *nose lick* and *lip corner puller*. However, this approach was shown to perform worse on long-lasting movements, such as *ears flattener*.

As DogFACS coding is a highly laborious and time-consuming task, automated movement recognition has the potential to enhance the process significantly. To demonstrate that, we developed a pilot DogFACS annotation model based on the trained event detectors. The results (41% reduction of mean annotation time per event) of our pilot with a certified DogFACS coder further confirm that this direction has the potential to reduce time and effort for the DogFACS coding process. One possible direction for further reducing the annotation time and cognitive load of the annotator could be by grouping DogFACS variables into facial parts (such as ears, eyes, and mouth) and displaying anomaly score plots for each region. Future work includes experiments with different ways to display the anomaly scores and their impact on annotation time and quality.

The limitation of the conducted case studies is the use of only one dataset, including a single breed. We chose to use it because, to the best of our knowledge, this is the only dog dataset where emotional states are experimentally induced in a very controlled way, with replicated results across samples and contexts. While we acknowledge that this may limit the generalisability of our findings, the choice was intentional as we know that differences in facial morphology can affect both the production and appearance of facial expressions in dogs. Therefore, focusing on a single, morphologically consistent breed represents a deliberate and systematic first step. By exploring facial behaviour within a well-characterized breed, we aim to establish a solid foundation before extending our methods to more morphologically diverse dog populations. Looking ahead, when using future datasets, it is important to carefully reflect on annotating emotional states and include the diversity of morphologies and appearances. In addition, we do not assert that the results from the two experiments are universal; rather, we consider them as preliminary findings that demonstrate the validity of landmark-based methods. For a more thorough investigation into emotion classification and the detection of FACS variables, further experiments are necessary. This should include exploring a variety of models, conducting hyperparameter searches, and performing extensive ablation studies, among other considerations.

Our main direction for future research is to improve the landmark pipeline, making it generic for all dog breeds and improving performance. With a deep learning approach, such an ambitious goal is hard to achieve, as we would need to include all dog breeds, ages, and appearances in the training set for such a model. But a landmark-based approach provides more flexibility, and including several morphological types, ear types, and cephalic types can be sufficient.

We hope that the DogFLW dataset will aid the scientific community in utilising AI-driven methods to deepen our understanding of our best friends’ behaviour and emotional world and investigate applications for veterinary healthcare, well-being, and sheltered and working dogs domains. In addition, we believe that datasets like DogFLW and CatFLW^58^ will play a critical role in promoting comparative research and advancing the broader application of affective computing in animal behaviour science.

## Supplementary Information

Supplementary Information.

## Data Availability

The DogFLW dataset will be available at https://github.com/martvelge/DogFLW at the moment of publication. The *Labrador Retriever* dataset is available from the original study of Bremhorst et al.^23^. The code generated during this study is available from the corresponding author upon reasonable request.
